# Emerging Clinical Features of COVID-19 Related Pancreatitis: Case Reports and Review of the Literature

**DOI:** 10.3389/fmed.2021.779118

**Published:** 2022-01-18

**Authors:** Vito Fiore, Rosalba Beretta, Andrea De Vito, Aleksandra Barac, Ivana Maida, David David Joeseph Kelvin, Claudia Piu, Vincenzo Lai, Giordano Madeddu, Salvatore Rubino, Goran Stevanovic, Stefan Korica, Sergio Babudieri

**Affiliations:** ^1^Unit of Infectious Diseases, Department of Medical, Surgical and Experimental Sciences, University of Sassari, Sassari, Italy; ^2^Department of Emergency, Area Socio-Sanitaria Locale, Olbia, Italy; ^3^Faculty of Medicine, University of Belgrade, Belgrade, Serbia; ^4^Clinic for Infectious and Tropical Diseases, University Clinical Center of Serbia, Belgrade, Serbia; ^5^Department of Microbiology and Immunology, Faculty of Medicine, Canadian Center for Vaccinology, Dalhousie University, Halifax, NS, Canada; ^6^Laboratory of Immunity, Shantou University Medical College, Shantou, China; ^7^Unit of Microbiology and Virology, Azienda Ospedaliero-Universitaria di Sassari, Sassari, Italy; ^8^Department of Biomedical Sciences, University of Sassari, Sassari, Italy; ^9^Faculty of Medicine, University of Belgrade, Belgrade, Serbia; ^10^Clinical Center of Serbia, University of Belgrade, Belgrade, Serbia; ^11^Clinic for Orthopaedic Surgery and Traumatology, University Clinical Center Serbia, Belgrade, Serbia

**Keywords:** COVID-19, SARS-CoV-2, severe pancreatitis, angiotensin-converting enzyme, acute

## Abstract

**Introduction:**

SARS-CoV-2 is fundamentally a respiratory pathogen with a wide spectrum of symptoms. The COVID-19 related pancreatitis is less considered than other clinical features. The purpose is to describe two cases of pancreatitis associated with COVID-19.

**Methodology:**

Patients' demographics, clinical features, laboratory, and instrumental findings were collected.

**Results:**

Two patients admitted to the hospital were diagnosed with COVID-19 and severe acute pancreatitis, according to the Atlanta criteria. Other causes of acute pancreatitis were excluded. Treatment included broad-spectrum antibiotics, proton pump inhibitors, and low molecular weight heparin. Steroids, oxygen, antifungal treatment, and pain killers were administered when appropriate. Both patients were asymptomatic, with normal vital parameters and blood exams, and were discharged in a good condition.

**Conclusion:**

It is recommendable to include lipase and amylase on laboratory routine tests in order to evaluate the need for the abdominal CT-scan and specific therapy before hospital admission of the patients with COVID-19 related life-threatening acute pancreatitis.

## Introduction

SARS-CoV-2 is fundamentally a respiratory pathogen with a wide spectrum of clinical features (1–5). Moreover, it can affect a few extra-pulmonary sights counting gastrointestinal tract, especially hepatocellular damage (6, 7). Angiotensin-converting enzyme-2 (ACE-2) receptor and transmembrane serine protease 2 (TMPRSS2), the section receptor for the causative coronavirus SARS-CoV-2 is more co-expressed within the gastrointestinal tract, hepatocyte, and cholangiocytes comparable to the respiratory mucosa. The presence of these receptors enables the passage of the virus into the tissue and causes coordinated tissue damage (8). This finding suggests how pancreatic injury may occur during COVID-19 and sporadic cases of COVID-19 related acute pancreatitis have been reported in the literature (9–13). However, the COVID-19 related pancreas involvement is less considered than other clinical features. Herein, we report two cases of COVID-19 with gastrointestinal symptoms as a clinical presentation of acute pancreatitis and mild respiratory involvement.

## Case 1

A 42-year-old man referred to the Emergency Department with a 3 days history of severe stabbing abdominal pain in the epigastric region with radiation to the back with frequent nausea and vomiting. The patient denied any other symptoms and epidemiological link with SARS-CoV-2 positive patients. He had no history of addiction, alcoholism, or medication. He was completely healthy a weak before admission, without any comorbidities. The patient had the following vital signs: heart rate 86 beats/min, blood pressure 140/100 mm/Hg and temperature 36.4°C, and O_2_ saturation 98% on room air. The abdomen was soft, mildly distended with severe epigastric tenderness and rebound tenderness. Laboratory workups showed high lipase (2,384 U/l) and amylase (416 U/l), elevated C reactive protein (CRP) (29 mg/dl), total bilirubin (2.28 mg/dl), glucose (131 mg/dl), D-dimer (0.97 mg/l), the blood count showed normal count of white blood cells (WBC) but increased neutrophils (83%) and decreased lymphocytes (10%) (Table 1). Ultrasound examination showed volumetric increase and hypo-echogenicity of the head of pancreas without gallbladder and Wirsung duct abnormalities. The patient underwent chest and abdomen computed tomography (CT) scan that revealed widespread bronchiectasis and signs of peri-bronchial and subpleural effusions in the posterior segment of the right lower lobe. In addition, CT revealed volumetric increase of the pancreatic gland with edema and irregular and poorly defined glandular contours, thickening of the retroperitoneal bands and mesenteric structures, presence of modest effusion both in the retroperitoneal space and in the peritoneal cavity, numerous enlarged reactive lymph nodes. No biliary ducts alterations or gallbladder stones were found. According to the hospital protocol, nasal and throat swabs were taken and polymerase chain reaction assay (PCR) showed positive result for SARS-CoV-2. According to the modified Atlanta criteria, the patient was diagnosed with severe pancreatitis and was admitted to the Infectious Diseases Department of the Sassari University Hospital, Sardinia, Italy.

**Table 1:** Laboratory findings of two patients with COVID-19 related acute pancreatitis.

**Parameter**	**Case 1**	**Case 2**
Glucose (mg/dl)	133	266
Total bilirubin (mg/dl)	2.28	1.1
Indirect (mg/dl)	1.42	–
Direct (mg/dl)	0.86	–
Amylase (U/l)	416	679
Lipase (U/l)	2,384	1,831
Calcium (mg/ml)	8.1	7.8
Albumin (g/l)	3.3	2.7
CRP (mg/dl)	28.57	15.38
PCT (ng/ml)	0.85	1.05
Hemoglobin (g/dl)	16.8	9.4
WBC count (cells/μl)	8,900	3,800
Neutrophil (%)	82.4	57.9
Lymphocyte (%)	10.5	32.6
D-dimer (mg/l)	0.97	1.92

*PCT, procalcitonin; CRP, C-reactive protein; WBC, white blood cell*.

Serology tests for Coxsackie virus, Epstein-Barr virus (EBV), and Cytomegalovirus (CMV) were negative. Patient was treated with broad-spectrum antibiotic (piperacillin/tazobactam 4.5 g 3x daily), low molecular weight heparin at prophylactic dose (4,000 UI/daily), proton pump inhibitor (pantoprazole 40 mg/daily), and painkillers when necessary. Patient did not need any ventilation support, PaO_2_/FiO_2_ ratio remained above 400 mmHg without need for O_2_ during hospital stay.

One week after the admission, PCR of the nasal and throat swab was repeated, and result came back as positive for SARS-CoV-2. After 31 days of hospital stay, the patient was discharged as asymptomatic, with normal vital parameters and blood exams, but the PCR for SARS-CoV-2 was still positive at discharge. The patient underwent to home isolation till negativity, which occurred 1 month after the discharge.

## Case 2

A 70-years-old man, with history of diabetes and chronic kidneys injury on dialysis, was admitted to a primary care hospital with a 10 days history of abdominal pain and dyspnea. Patient was febrile (38.5°C), hypotensive (blood pressure 90/60 mmHg), with 91% O_2_ saturation. The patient had no history of addiction, alcoholism, or medication. Blood gas analysis showed mild ARDS type 1, with PaO_2_/FiO_2_ ratio 268 mmHg. No alterations were found in electrocardiography, troponin, and cardiological examinations, but biochemical laboratory findings were suggestive for pancreatitis. Laboratory findings are summarized in Table 1. The patient underwent chest and abdomen CT scan that revealed bilateral interstitial pneumonia compatible with COVID-19 (lung score = 5), edematous pancreas with blurred and edematous margins, widespread imbibition of peripancreatic adipose tissue, and anterior renal bands inflammatory suffusion. Biliary ducts, Wirsung, and gallbladder did not show alterations or stones. Given the respiratory situation and the lung CT-scan, PCR on nasopharyngeal swab for SARS-CoV-2 was performed and results came back as positive. The patient was transferred to the Infectious Diseases Department of the Sassari University Hospital, Sardinia, Italy.

Serology tests for Coxsackie virus, EBV, and CMV were negative. Treatment included broad-spectrum antibiotic (piperacillin/tazobactam 4.5 g 3x daily), caspofungin (Ostrosky-Zeichner score indicative for high risk of invasive candidiasis) (14), low molecular weight heparin at prophylactic dose (4,000 UI/daily), proton pump inhibitor (pantoprazole 40 mg/daily), dexamethasone 6 mg/daily for 10 days, and O_2_ supplement. Insulin dosage was adapted according to glycemia and 3 times weekly dialysis was also prosecuted. Remdesivir was not administrated in according to the national guidelines, due to the kidneys' injury. The patient was discharged after 40 days, in good health condition, normalized biochemical laboratory parameters, good oxygen saturation and negative PCR test for SARS-CoV-2.

## Discussion

Viral pancreatitis has been well-described in the literature, most commonly related to mumps, measles, coxsackie, EBV, CMV, and Hepatitis-A virus (HAV) (15). As reported by Brickman and Spinelli, COVID-19 clinical features may include pancreatitis (16, 17). Acute pancreatitis in COVID-19 could occur due to the direct cytopathic effect of local SARS-CoV-2 replication or indirectly, by the viral-induced immune response (18). Literature shows that 17% of patients with severe COVID-19 have pancreatic injury with high serum lipase and amylase levels, while 7% of them had imaging findings compatible with pancreatitis (19). In the literature, onset of the COVID-19 related pancreatitis is usually late. Hadi described a case of three patients from the same family, hospitalized due to the COVID-19 related acute pancreatitis (10). Anand et al. reported a case of a 59-year-old woman with COVID-19, who had acute pancreatitis immediately after hospital admission due to the COVID-19 related bacterial pneumonia (12).

Our patients presented acute pancreatitis as onset of COVID-19 symptoms: severe acute pancreatitis in according with the Atlanta criteria (abdominal pain, increased serum lipase level to greater than 3 times the upper limit of normal value and confirmed acute pancreatitis on CT-scan) (20).

Concurring to the present findings, the acute abdomen is one of the appearances of COVID-19 infection, and different causes such as acute pancreatitis should be on the list of differential diagnoses. Timely diagnosis may have a noteworthy effect on the patient's treatment. It is recommendable to include lipase and amylase on laboratory routine test in order to evaluate need for the abdominal CT-scan and specific therapy before hospital admission of the patients with COVID-19 related life-threatening acute pancreatitis.

## Limitations of the Study

Some limitations should be acknowledged in regard of our study. First of all, it is retrospective and limited to two cases. More studies with large sample size and prospective construction are needed to confirm our data. For now, COVID-19 as the cause of acute pancreatitis has not been established yet due to insufficient evidence (11). In Italy, the most prevalent cause of acute pancreatitis is the biliary tract obstruction (21). CT scan is described as a method with variable accuracy when evaluating choledocholithiasis, with sensitivity and specificity in a range of 60–87% and a of 97–100%, respectively (22, 23). Furthermore, microlithiasis remains one of the causes of biliary pancreatitis and should be evaluated either by magnetic resonance cholangiopancreatography (MRCP) or endoscopic ultrasound (EUS), given the CT scans' lower accuracy. However, the use of MRCP and EUS among patients with diffusive infectious diseases is limited in our center for security reasons.

## Strength of the Study

Although some limitations were present, the diagnosis of acute pancreatitis in both cases was established using a complete set of data, including clinical features, laboratory findings and imaging with CT scan.

## Data Availability Statement

The original contributions presented in the study are included in the article/supplementary material, further inquiries can be directed to the corresponding author/s.

## Ethics Statement

Ethical review and approval was not required for the study on human participants in accordance with the local legislation and institutional requirements. The patients/participants provided their written informed consent to participate in this study. Written informed consent was obtained from the individual(s) for the publication of any potentially identifiable images or data included in this article.

## Author Contributions

VF, RB, AD, AB, and SB designed the manuscript and wrote the first draft. IM,DJ, CP, VL, and GM did review of the literature. SR, SK, and GS did critical revision of the manuscript. All authors contributed to manuscript conceiving, drafting, and approved the final version.

## Funding

This work was supported by awards from the Canadian Institutes of Health Research (CIHR OV2−170357), Research Nova Scotia, Atlantic Genome/Genome Canada, Li-Ka Shing Foundation, and Dalhousie Medical Research Foundation (DJ). DJ was a recipient of a Canada Research Chair in Translational Vaccinology and Inflammation.

## Conflict of Interest

The authors declare that the research was conducted in the absence of any commercial or financial relationships that could be construed as a potential conflict of interest.

## Publisher's Note

All claims expressed in this article are solely those of the authors and do not necessarily represent those of their affiliated organizations, or those of the publisher, the editors and the reviewers. Any product that may be evaluated in this article, or claim that may be made by its manufacturer, is not guaranteed or endorsed by the publisher.
